# Physico-mechanical and dissolution behaviours of ibuprofen crystals crystallized in the presence of various additives

**Published:** 2010

**Authors:** A. Nokhodchi, O. Amire, M. Jelvehgari

**Affiliations:** 1 Chemistry and Drug Delivery Group, Medway School of Pharmacy, Universities of Kent and Greenwich, Kent, UK; 2 Drug Applied Research Centre and Faculty of Pharmacy, Tabriz University of Medical Sciences, Tabriz, Iran

**Keywords:** Crystallization, Ibuprofen, Crystal habit, Additives, Tensile strength, Dissolution, Solid state

## Abstract

**Background and the purpose of the study:**

The success of any direct-tableting procedure is strongly affected by the quality of the crystals used in the process. Ibuprofen is a poorly compactible drug with a high tendency for capping. In order to use ibuprofen in direct compression formulations, physico-mechanical properties of ibuprofen should be improved considerably. The aim of the present investigation was to employ crystallization techniques in order to improve the physico- mechanical properties of ibuprofen for direct compression.

**Methods:**

The experimental methods involved the preparation of ibuprofen crystals by solvent change technique. Ibuprofen was dissolved in ethanol and crystallized out with water in the absence or presence of various hydrophilic additives (PEG 6000, 8000, Brij 98P and polyvinyl alcohol 22000, PVA _22000_) with different concentrations. The physico-mechanical properties of the ibuprofen crystals were studied in terms of flow, density, tensile strength and dissolution behaviour. Morphology of ibuprofen crystals was studied by scanning electron microscopic (SEM). Solid state of the recrystallized particles was also investigated using differential scanning calorimeter (DSC) and FT-IR.

**Results:**

Ibuprofen samples crystallized in the presence of PEG 6000 and 8000 and PVA showed remarkable increase in the tensile strengths of the directly compressed tablets, while some other additives, i.e. Brij 98P did not produce improved ibuprofen crystals. Ibuprofen powders made from particles obtained in the presence of PVA and Brij 98P showed similar dissolution profiles to the commercial ibuprofen particles. DSC and FT-IR results ruled out any significant interaction between ibuprofen and additives except for the samples crystallized in the presence of PEG 8000.

**Conclusion:**

The crystal habit of ibuprofen can be altered successfully by the crystallization technique which was developed in this study. The crystals developed in the presence of certain additives can be recommended for direct compression.

## INTRODUCTION

Crystallization is a major technological process for particle formation in the pharmaceutical industry. Many drug substances form different crystal lattices with different internal packing arrangements of molecules; thus polymorphic forms exist. However, there are also drugs whose crystals only have differences in their outer appearance; these drugs form different habits without differences in the crystal lattice (crystal habit). The crystal habit of a drug is an important variable in pharmaceutical manufacturing. Different crystal forms of a particular drug possess different planes and thus differ not only in their specific surface, but also in their free surface energy. Therefore, they may exhibit different physico-mechanical properties (1). It has been shown that properties such as dissolution rate, powder flow, and compressibility, which are of pharmaceutical interest, can differ for different habits of the same drug (2–5). For example, acetaminophen can exist in two polymorphic forms, the common crystal form is the thermodynamically stable form I (monoclinic) which leads to unstable tablets with high capping tendency due to a stiff construction of the molecules inside the crystal and form II (orthorhombic) that shows better compaction behaviour (6).

Ibuprofen a common analgesic drug with antipyretic action is widely administered orally and topically (7). It is also used in the relief of symptoms of rheumatoid arthritis and osteoarthritis (8). Because of its hydrophobic properties, it shows low solubility in water. Ibuprofen shows poor compressibility and compactibility under compaction process and most of the tablets produced at high speed were capped (9), thus in most cases for production of solid dosage forms a granulation step is necessary. Ibuprofen can form different crystal forms with different properties. It has been shown that the type of solvent and the presence of a methacrylic polymer had remarkable effect on the crystal habit of ibuprofen prepared by a temperature change method (10). Crystallization was carried out with different cooling rates using different alcohols with acetone in the presence of different methacrylic polymers. They showed that the solvent and the cooling rate had an effect on the micromeritic properties due to interactions with hydrophobic faces of the growing crystals, but the polymer had a smaller effect (10). The polarity of the solvent affects the crystal habit, as the use of methanol resulted in the most symmetrical crystals while the use of a low polarity solvent (acetone) resulted in elongated crystals. A further study (11) described differences in crystal habit of ibuprofen crystallized by a cooling process from different alcohols and hexane. During the cooling process ibuprofen powder was added as nuclei. While ibuprofen crystallized from methanol and ethanol shows a polyhedral crystal habit, crystallization from hexane resulted in needle-like crystals.

Lee et al (12) used different solvents to crystallize ibuprofen and acetaminophen and showed that in order to have a successful scale up preparation, a full knowledge of solubility, polymorphism, crystal habit and crystallinity of solid compounds is necessary, however they did not study the effect of the type of solvent on mechanical behaviour and dissolution of ibuprofen powders. Similar study was carried out by Gavrilin et al., showed the solubility of ibuprofen was affected by the type of crystal habit (13). The work published by Di Martino was mainly about densification mechanism of ibuprofen crystallized from different organic solvents (14). The effect of various additives on micromeritic, dissolution and mechanical behaviours of crystallized ibuprofen powders has not been described previously.

Ibuprofen is presently being crystallized in the commercial scale from hexane and heptan into a crystal that has a poor flow and compaction properties owing to its needle-like crystalline and viscoelastic property (15, 16) which creates great difficulties in direct compression formulations. Therefore the aim of the present investigation was to explore a suitable solvent and additive for crystallization of ibuprofen in order to improve its physic-mechanical properties for direct compression purpose without any negative effect on its dissolution performance.

## MATERIALS AND METHODS

Ibuprofen (Spectrum, USA), ethanol (Fisher, UK), Polyethylene glycol (PEG) with molecular weight of 6000 and 8000 (Acros Organics, USA), Brij 98P (Sigma-Aldrich, USA) and polyvinyl alcohol (PVA) with molecular weight of 22000 (Acros Organics, USA) were used in this investigation.

### 

#### Scanning electron microscope (SEM)

The shape and surface topography of crystallized and commercial ibuprofen crystals were determined by using scanning electron microscope (LEO 440I, Cambridge, UK) operating at 15 kV. The specimens were mounted on a metal stub with double-sided adhesive tape and coated under vacuum with gold in an argon atmosphere prior to observation.

#### Differential scanning calorimetery (DSC)

Samples of the crystals (about 5 mg) were heated (30–120 °C) at a scanning rate of 10 °C/min in crimped hemetically-sealed aluminium pans under a nitrogen atmosphere. The enthalpy of fusion and melting point could be obtained from the thermograms using the instrumental software (DSC 822 Mettler Toledo, Switzerland). The calorimeter was calibrated using indium and lead standards.

#### Fourier transforms infrared spectroscopy (FT-IR)

A computerized FT-IR (PerkinElmer, USA) was used to obtain the spectrum of various ibuprofen samples. The crystal sample (about 10 mg) was placed on the plate of the machine and the handle was placed on powder sample to generate enough pressure for compression. The spectrum for each sample showed the wavelength of absorbed light which is a characteristic of the chemical bonds in the sample. The scanning range was 600–4000 cm^−^1 and the resolution was 1 cm^−1^.

#### Compressibility index measurement

Flowability of untreated and agglomerated samples was also assessed from Carr's Index (CI) (17, 18). The CI was calculated from the poured and tapped densities. Tapped density was determined by tapping the samples (10 g) into a 10 ml measuring cylinder using a tapping machine. The CI was calculated according to the following equation.

1 CI=[(Tapped density-Bulk density)/Tapped density]×100

#### Pressure-tensile strength relationship

The ibuprofen particles (100±5 mg) were compressed using a 6 mm flat-faced punch at a constant compression speed with different compaction pressures (50, 100 and 150 MPa), using a hydraulic press (Minipress, TP3, Copley, UK). Lubrication of the die and punch was performed using a 1% w/v dispersion of magnesium stearate in acetone. The compacts were allowed to relax for 24 hrs and the force fracturing the compact (F) was measured. The tensile strength (T) of the compact was calculated according to the following equation (19).T=2F/ (πDt)

where D and t are the diameter and thickness of the compact, respectively. The results are the mean and standard deviations of a minimum of 5 determinations.

#### Dissolution test

The ibuprofen powder (100±10 mg) was filled into capsules without any additional excipient. Dissolution was performed (Erweka DT700, Germany) in 900 ml of phosphate buffer of pH 7.2 at 50 rpm and 37±0.1°C using the rotating paddle method. Samples of the solution were withdrawn at pre-determined time intervals (3, 6, 9, 12, 15, 20, 25,30, 40, 50 and 60 min) using peristaltic pump attached to the dissolution tester. The amount of dissolved ibuprofen was analyzed spectrophotometrically (UV-160, Shimadzu, Japan) at 221 nm.

## RESULTS AND DISCUSSION

### 

#### Micromeritic and morphology studies of ibuprofen crystals

**Figure 1:**
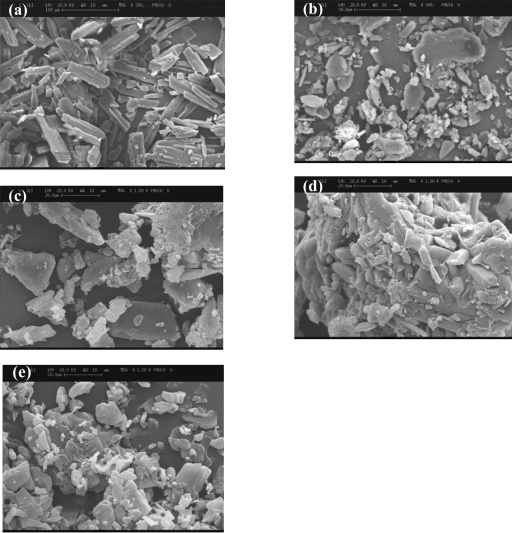
Scanning electron micrographs of (a) untreated, and treated ibuprofen powders in: (b) absence of additives, (c) presence of 5% PEG 8000, (d) presence of 5% PEG 6000, (e) presence of 5% PVA_22000_.

It has already been shown that the crystal habit of ibuprofen depends on crystallization conditions such as the type of solvent and the presence of additives (11, 16, 20–22). SEM of various recrsytallized ibuprofen samples are shown in figure 1. The figure shows that the commercial ibuprofen is needle- like shaped, whereas the samples recrysallized in the presence of additives showed plate-like (figure 1c) or irregular shape (figure 1b and e). When crystallization was carried in the presence of PEG 6000, agglomerated particles were observed (figure 1d). As a result of the different crystal morphology, the prepared ibuprofen crystals might show significant differences in the micromeritic and mechanical properties. Micormeritic behaviours of the commercial ibuprofen and all engineered ibuprofen particles are listed in table 1. It is obvious from Carr's index values that the flow of commercial ibuprofen is extremely poor due to a high cohesivity and adhesivity. Because of poor flowability and compactibility of the commercial ibuprofen powder, in most cases the drug has to be granulated before tableting. The micrormeritic properties of ibuprofen can be improved by choosing a suitable crystal form (a suitable crystal habit). Table 1 shows that all engineered ibuprofen powders showed lower Carr's index than the commercial ibuprofen powder which is an indication of an improvement in flow behaviour of ibuprofen powder. The increase in powder flow of engineered particles could be partly due to an increase in true density of ibuprofen powders (Table 1). Table 1 also shows the bulk density data of various ibuprofen samples. The table shows that, in general, the best flow is obtained for the samples with high bulk density. The samples with bulk density above 0.35 g/cm^3^ showed better flowability than the samples that their bulk densities are smaller than 0.35 g/cm^3^. The differences in the bulk density of various ibuprofen samples may be related to their markedly different crystal habits, leading to different contact points and frictional and cohesive forces between the crystals. These factors in turns affect the sliding of the particles against each other, leading to different packing geometry and hence different bulk densities. These data are in good agreement with morphology of ibuprofen crystals (Figure 1) since the commercial ibuprofen shows needle-like crystals hence low bulk density and low flowability. The study showed that the presence of additive in the crystallization medium can improve flow of ibuprofen powders. Similar results has been reported by Rasenack and Muller (22), showing that when ibuprofen is crystallized in the presence of hydrophilic additives such as sucrose, an improvement in flow of powder is achieved. As it is shown in figure 1 ibuprofen which crsytallized in the presence of additives showed different crystal shapes and in the case of samples which were recrystallized in the presence of PEG 6000 some agglomerations were observed. Rasenack and Muller (22) also showed that ibuprofen particles can be agglomerated if it is recrystallized from acetonitrile and methanol. In another study they showed that the shape of crystals can be modified when ibuprofen was crystallized in the presence of various hydrophilic additives (16). The properties of the additives such as interaction with the dissolved ibuprofen might be the main factor that influences the direction in which crystals grow on the nuclei. Therefore, the formation of different crystal shapes in the presence of different additives may be attributed to the interactions of these additives with ibuprofen.

**Table 1:** Micromeritic properties of various ibuprofen crystals (the results are the mean and standard deviations of 3 determinations).

Sample	True density (g/cm^3^)	Bulk density (g/cm^3^)	Carr's Index (%)
Commercial ibuprofen	1.085±0.027	0.25±0.03	45.0±2.5
Engineered ibuprofen without additive	1.110±0.003	0.38±0.03	26.3±2.2
Engineered Ibu. 1%PEG 8000	1.132±0.002	0.36±0.05	27.8±3.6
Engineered Ibu. 2%PEG 8000	1.222±0.008	0.52±0.03	27.5±2.5
Engineered Ibu. 5%PEG 8000	1.139±0.015	0.35±0.02	23.8±1.8
Engineered Ibu. 1%PEG 6000	1.161±0.005	0.29±0.06	24.4±2.2
Engineered Ibu. 2%PEG 6000	1.158±0.003	0.38±0.04	27.5±2.6
Engineered Ibu. 5%PEG 6000	1.1430.004	0.39±0.04	28.9±3.1
Engineered Ibu. 1% Brij 98P	1.148±0.005	0.28±0.02	34.9±3.2
Engineered Ibu. 2% Brij 98P	1.150±0.003	0.32±0.04	34.2±3.0
Engineered Ibu. 5% Brij 98P	1.146±0.001	0.36±0.02	27.9±1.7
Engineered Ibu. 1% PVA_22000_	1.014±0.006	0.30±0.06	40.0±4.3
Engineered Ibu. 2% PVA_22000_	1.259±0.019	0.24±0.05	32.5±3.1
Engineered Ibu. 5% PVA_22000_	1.185±0.005	0.25±0.02	33.3±1.9

#### Mechanical properties of crystallized ibuprofen samples

**Figure 2:**
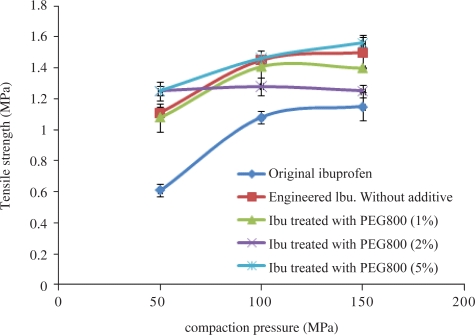
The effect of compaction pressure on tablets made from engineered ibuprofen particles in the presence of PEG 8000 (the results are the mean and standard deviations of 6 determinations).

**Figure 3:**
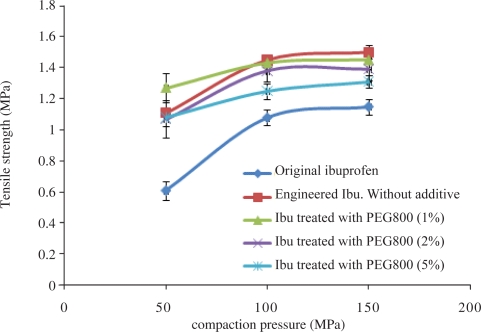
The effect of compaction pressure on tablets made from engineered ibuprofen particles in the presence of PEG 6000 (the results are the mean and standard deviations of 6 determinations).

**Figure 4:**
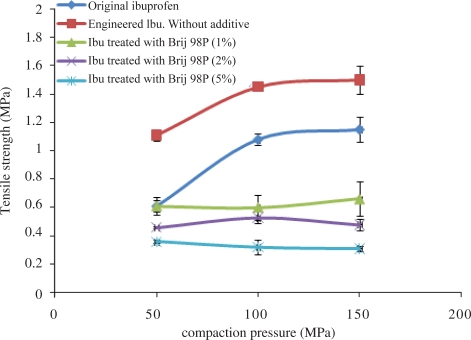
The effect of compaction pressure on tablets made from engineered ibuprofen particles in the presence of Brij 98P (the results are the mean and standard deviations of 6 determinations).

**Figure 5:**
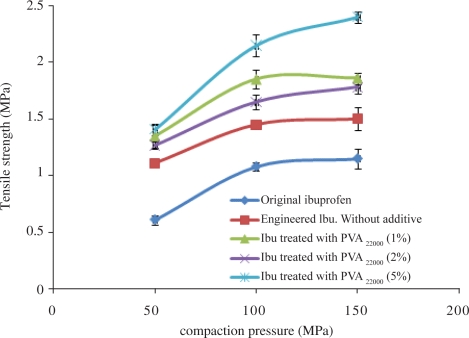
The effect of compaction pressure on tablets made from engineered ibuprofen particles in the presence of PVA_22000_ (the results are the mean and standard deviations of 6 determinations).

**Figure 6:**
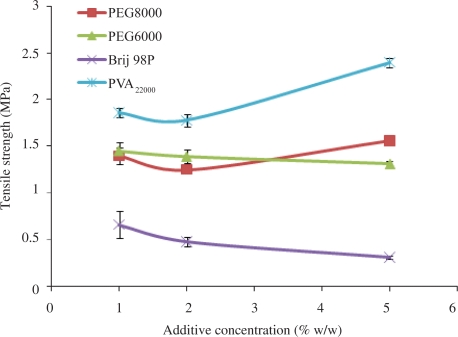
The effect of additive concentration on tensile strength from ibuprofen tablets made of various engineered particles (the results are the mean and standard deviations of 6 determinations).

Compactibility or tabletability, which is the capacity of a powdered material to be transformed into a tablet of a specific strength under the effect of compaction pressure (24) are presented in (figures 2–5). In these figures the tensile strength of all tablets made from various ibuprofen crystals obtained in the presence of various additives with different concentrations were plotted against compaction pressure. Two-way analysis of variance showed that with exception of tablets made from ibuprofen crystals obtained in the presence of Brij 98P, the type of additives and compression pressure had significant effect on tensile strength of tablets made from ibuprofen crystals (Figure 4). It is obvious from all these figures that tablets made from engineered ibuprofen crystals in most cases showed superior compactibility than the commercial ibuprofen crystals. In the case of ibuprofen recrystallized in the presence of various concentration of PEG 8000, all recrystallized samples showed higher tensile strength than untreated ibuprofen. Similar results may be drawn for those samples recrystallized in the presence of PEG 6000 (Figure 3) and PVP_22000_ (Figure 5). However in the case of Brij 98P only the recrystallized ibuprofen in the absence of additive showed higher tensile strength than untreated ibuprofen (Figure 4). In other words, ibuprofen recrystallized in the presence of various concentrations of Brij 98P showed weaker tablets than the commercial ibuprofen tablets. To compare the effect of various additives with the same concentration on ibuprofen tablets figure 6 was constructed. The figure shows that ibuprofen tablets made of recrystallized drug in the presence of PVA showed superior tensile strength than other additives. This indicates that the effect of additives on tensile strength of ibuprofen tablets can be ranked as PVA>PEG6000=PEG 8000>Brij 98P. The results showed that when the molecular weight of PEG was increased from 6000 to 8000, no significant changes (p>0.05) were observed for the tensile of ibuprofen tablets (Figure 6). The poor compactibility of drugcrystals may be attributed to the presence of crystal faces that give poor adhesion to other crystals and the absence of the faces that are required for optimal adhesion (24). The poor compactibility of crystals could partly be due to morphology of ibuprofen crystals (Figure 1). It has been shown that in the case of ibuprofen needle-like and grain-like crystals the relative abundance of the different faces within the crystals was modified (11). This can affect the inter-particulate bonding between these crystals, resulting in different compaction properties. Figure 6 also showed that the concentration of additive used in the crystallization process of ibuprofen had no remarkable effect on the tensile strength of ibuprofen tablets.

#### Dissolution study

**Figure 7:**
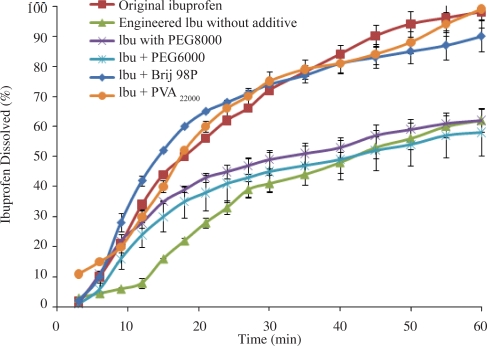
Dissolution behaviour of various ibuprofen crystals (the results are the mean and standard deviations of 3 determinations).

Ibuprofen is poorly water soluble drug and belongs to class II drug. Therefore an increase in the dissolution rate of ibuprofen should increase its bioavailability. The dissolution profiles of ibuprofen crystallized in the presence of various additives shown in figure 7 reveals that samples crystallized in the absence of additive and in the presence of 5% w/v PEG 8000 and PEG 6000 showed poorer dissolution performance than the commercial ibuprofen. On the other hands, ibuprofen samples crystallized in the crystallization medium had no significant effect on ibuprofen dissolution, so, these figures were not included in the present study. In summary, it may be concluded that PVA is the best additive to be added to ibuprofen crystallization medium to produce robust tablet with at least similar dissolution performance as untreated ibuprofen samples.

#### Solid state studies

**Figure 8:**
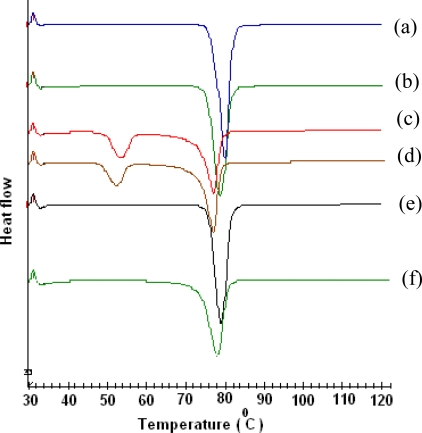
Scanning electron micrographs of (a) untreated, and treated ibuprofen powders in: (b) absence of additives, (c) presence of 5% PEG 8000, (d) presence of 5% PEG 6000, (e) in the presence of 5% Brij 98P, (f) presence of 5% PVA_22000_.

DSC was used to investigate any change in the type of polymorph during the crystallization between presence of Brij 98P and PVA additives and ibuprofen. The DSC thermograms of the same performance as untreated ibuprofen. The results showed that the concentration of additives in untreated ibuprofen and all ibuprofen crystallized in the presence of various additives are shown in figure 8. Since all thermograms were similar, only the thermograms of the samples crystallized in the presence of 5% additive are shown. The enthalpy and melting point of all samples are reported in table 2. The results showed that the commercial tab ibuprofen had a sharp endothermic peak around 79 °C indicating the fusion of ibuprofen at this temperature with an enthalpy of 128.75 J/g. The thermogram of recrystallized ibuprofen without any additive is similar to the commercial ibuprofen with a small reduction in the fusion point. The statistical test showed that the reduction in the fusion and enthalpy are not significantly different compared to the commercial sample (P>0.05).

**Table 2:** Thermal behaviour of various ibuprofen samples (the results are the mean and standard deviations of 3 determinations).

Sample	Melting point (°C)	Enthalpy (J/g)
Commercial ibuprofen	78.46±1.62	128.75±1.0
Engineered ibuprofen without additive	79.18±1.37	126.55±1.3
Engineered Ibu. 1%PEG 8000	77.10±1.21	95.4±2.5
Engineered Ibu. 2%PEG 8000	76.95±1.15	90.2±2.1
Engineered Ibu. 5%PEG 8000	76.45±1.11	83.3±1.2
Engineered Ibu. 1%PEG 6000	77.95±1.90	108.1±2.6
Engineered Ibu. 2%PEG 6000	77.56±1.84	100.1±2.4
Engineered Ibu. 5%PEG 6000	77.48±1.50	88.0±1.9
Engineered Ibu. 1% Brij 98P	78.20±1.24	119.4±2.5
Engineered Ibu. 2% Brij 98P	78.02±1.20	115.2±3.1
Engineered Ibu. 5% Brij 98P	78.00±0.80	107.1±2.1
Engineered Ibu. 1% PVA_22000_	78.40±1.33	129.1±1.9
Engineered Ibu. 2% PVA_22000_	78.45±1.9	127.2±1.6
Engineered Ibu. 5% PVA_22000_	79.68±1.86	124.5±2.9

This indicates that the crystallization of ibuprofen from ethanol had no significant effect on ibuprofen polymorph. The thermograms of ibuprofen samples crystallized in the presence of different PEGs showed two endothermic peaks related to the fusion of PEG and melting of ibuprofen respectively. This indicates that PEG molecules might be entrapped or adsorbed to the ibuprofen crystals during the crystallization process. It is clear from the table that as more PEG was added to the crystallization medium the enthalpy of ibuprofen peak decreased which could be due to the presence of more PEG molecules adsorbed to ibuprofen samples at high concentration of additives. Another reason for the reduced enthalpy of ibuprofen peak could be due to the dissolution of ibuprofen particles in the melted PEG during the heating. These could be the main reasons why ibuprofen melting point is slightly shifted to lower temperatures for samples. All these data indicate that no polymorphic modification occurred during the crystallization of ibuprofen particles.

**Figure 9:**
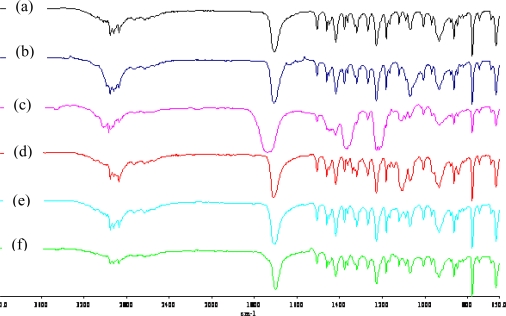
FT-IR spectra of (a) untreated, and treated ibuprofen powders in: (b) absence from additives, (c) presence of 5% PEG 8000, (d) in presence of 5% PEG 6000, (e) presence of 5% Brij 98P, (f) presence of 5% PVA_22000_.

Further analysis was carried out by FT-IR to identify any changes in molecular level. The FT-IR spectra of ibuprofen showed characteristic peaks at 1710 cm^−1^ and 2920 cm^−1^ due to carbonyl and hydroxyl stretching respectively (Figure 9). These characteristic peaks appeared in all FT-IR spectra of crystallized ibuprofen samples indicating no changes in molecular level of ibuprofen when it is recrystallized in the presence of additives. However, in the case of the sample which was crystallized in the presence of PEG 8000, the peak at 1710 cm^−1^ was shifted to higher wave number indicating interaction between ibuprofen and PEG 8000. Such an interaction did not occur for ibuprofen samples obtained in the presence of PEG 6000.

## CONCLUSION

Crystal habit of ibuprofen was successfully modified by crystallization of the drug in the presence of various hydrophilic additives. The results showed that the crystal habit modification of ibuprofen improves the flow of powder and also mechanical behaviour of ibuprofen tablets without any negative effect on its dissolution performance. The crystals which developed in the presence of certain additives can be recommended for direct compression.
